# The Potentials of Methylene Blue as an Anti-Aging Drug

**DOI:** 10.3390/cells10123379

**Published:** 2021-12-01

**Authors:** Huijing Xue, Abhirami Thaivalappil, Kan Cao

**Affiliations:** Department of Cell Biology and Molecular Genetics, University of Maryland, College Park, MD 20742, USA; hjxue05@umd.edu (H.X.); ami.thaivalappil@gmail.com (A.T.)

**Keywords:** methylene blue, mitochondria, neurodegeneration, skin aging, progeria

## Abstract

Methylene blue (MB), as the first fully man-made medicine, has a wide range of clinical applications. Apart from its well-known applications in surgical staining, malaria, and methemoglobinemia, the anti-oxidative properties of MB recently brought new attention to this century-old drug. Mitochondrial dysfunction has been observed in systematic aging that affects many different tissues, including the brain and skin. This leads to increaseding oxidative stress and results in downstream phenotypes under age-related conditions. MB can bypass Complex I/III activity in mitochondria and diminish oxidative stress to some degree. This review summarizes the recent studies on the applications of MB in treating age-related conditions, including neurodegeneration, memory loss, skin aging, and a premature aging disease, progeria.

## 1. Introduction

**Figure 1:**
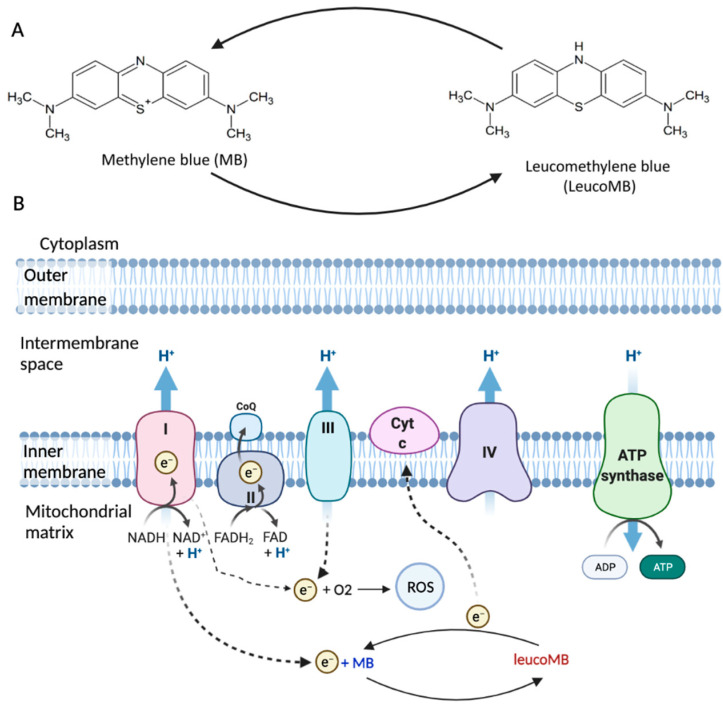
The structure and function of MB. (**A**) MB is a phenothiazine derivative, and its reduced form is leucoMB. (**B**) Mitochondrial electron transport chain (ETC) is associated with ATP production and ROS production. MB can work as a catalytic redox cycler in mitochondria and bypass Complex I/III activity (Figure adapted from “Electron Transport Chain”, by BioRender, 10 October 2021, retrieved from https://app.biorender.com/biorender-templates/t-5edadc578a691400acf3c4ec-electron-transport-chain).

Methylene blue (MB) is a well-established drug, originally synthesized as a textile dye in 1876 [1]. The small molecular weight allows MB to be delivered into tissues fast. MB can be reduced into leucomethylene blue (leucoMB), and therefore, it can be used as an antioxidant (Figure 1A) [2]. MB is an FDA-approved medicine and has been widely used in surgical staining, malaria, methemoglobinemia, etc. [1].

According to the “free radicals theory of aging”, cell and tissue aging are a result of free radicals’ attacks [3]. In human cells, reactive oxygen species (ROS) are mainly produced in mitochondria [4], while aging, declines in mitochondrial mass, respiration capacity, and respiration efficiency were observed in different tissues [5]. Dysfunctional mitochondria lead to decreased ATP production and increased ROS production [6], which would further damage mitochondria. This vicious cycle leads to severe cellular damages and accelerates the aging process. MB’s antioxidative properties mainly improve mitochondrial function and break the ROS–mitochondrial damage cycle [7,8], which makes it a great candidate as an anti-aging drug.

In this review, the structure, biological function, and applications of MB will be discussed.

## 2. Structure and Functions

A mitochondrion is a major energy source in eukaryotic cells, and the mitochondrial electron transport chain (ETC) is associated with ATP production [9]. ETC is a series of electron transporters located in the inner mitochondrial membrane that shuttles electrons from NADH and FADH2 to molecular oxygen (Figure 1B). ETC consists of four complexes, with Complex I (NADH-ubiquinone oxidoreductase) and Complex II (succinate dehydrogenase) being the entrances for electrons, and NADH and FADH_2_ transfer the electrons to Complex I and Complex II, respectively [10]. These electrons are transported by Complexes III (CoQ-cytochrome c reductase) and IV (cytochrome c oxidase), with the help of ubiquinone (Co-enzyme Q10, CoQ) and cytochrome c. Complex III passes the electrons to cytochrome c, and Complex IV transfers the electrons from cytochrome c to the terminal electron acceptor O_2_ to generate H_2_O [11]. In this process, Complex V (ATP synthase) will produce ATP by phosphorylating ADP. About 0.4% to 4% oxygen is partially reduced under physiological conditions, with ROS being the byproduct [12]. There are 11 sites of ROS generation in the ETC, however, it is mainly produced in Complex I [13,14]. Mitochondrial dysfunction can lead to oxidative damage, which primarily impairs Complex IV as well as Complex I [15]. While Complex I is impaired, overproduction of ROS can result in increased oxidative stress and therefore induce cellular damages [4].

MB is a phenothiazine derivative and can be reduced into leucoMB (Figure 1A). In its oxidized state, MB solution is blue, while leucoMB is colorless [16]. MB is hydrophilic and lipophilic, which makes it highly permeable through biomembranes [17]. MB is also positively charged [18]. The redox potential of MB is 11 mV, and it can cycle readily between oxidized and reduced forms in mitochondria because of its low redox potential [8]. Such characteristics allow MB to work as a catalytic redox cycler in mitochondria, promoting cytochrome oxidase activity and ATP production (Figure 1). MB also decreases the production of ROS via bypassing Complex I/III activity [18,19]. MB receives electrons from NADH through Complex I, converting to leucoMB. LeucoMB can directly transfer these electrons to cytochrome c, re-oxidized to MB. Therefore, MB has the potential to protect cells against oxidative stress under pathological conditions.

MB has been widely used in surgical staining, malaria, and methemoglobinemia [1]. Additionally, MB was shown to photo-inactivate bacteria in 1928 [20], and its potent antiviral effects were discovered soon after [21]. MB can also be used as an anti-fungal and anti-parasitic aquarium disinfectant, even with very high dosages (millimolar) [22,23]. MB has also been used in photodynamic therapy among different types of cancers, including lung cancer [24], breast cancer [25], and prostate cancer [26]. Surprisingly, a French study involving 2500 patients treated with MB and standard chemotherapy showed no cases of COVID-19 infection [27]. A study showed that low doses (0.5–4 mg/kg) of MB are effective to stimulate mitochondrial respiration in vivo and safe in animals and humans [17]. Such evidence suggested that MB is a safe drug.

## 3. Applications

### 3.1. MB in Brain Aging

Oxidative metabolism is an important energy source for brain activity [28]. During brain aging, mitochondrial dysfunction has been associated with neuronal loss. It has also been observed in many brain diseases, such as Alzheimer’s disease (AD), Parkinson’s disease (PD), and brain injuries [29]. MB is highly lipophilic and able to effectively cross the blood-brain barrier (BBB) [17]. MB concentration is found to be higher in the brain than in plasma after oral administration or intravenous injection in rats [30]. Besides, it has a strong affinity for mitochondria [31]. Different from the other antioxidants such as MitoQ and MitoVitE, MB can reduce the production of free radicals by bypassing Complex I/III activity rather than scavenging free radicals [32]. In fact, the membrane potential in Complex III-inhibited mitochondria can be partially restored by MB in both mice and rats [18]. MB, acting as an electron donor, can also increase the expression of brain cytochrome oxidase and oxygen consumption in vivo [33,34]. Besides, low doses of MB were shown to effectively inhibit nitric oxide (NO), which inhibits cytochrome c oxidase activity [35,36]. All these properties make MB a promising drug candidate for brain diseases’ treatment.

AD is a neurodegenerative disease that constitutes a significant portion of dementia [37]. Amyloid-β (Aβ) aggregation and neurofibrillary tangles (NFTs) are two pathological hallmarks of AD [38]. Aging is one of the most important risk factors of AD [39]. Mitochondrial dysfunction could be a missing link between aging and AD: at the early stage of AD progression, elevated mitochondria-derived oxidative stress has been reported [40,41]. Mitochondria with reduced size and impaired movement were also observed in the AD brain [42]. Moreover, mitochondrial dysfunction causes diminished energy metabolism, alterations in the key enzymes in oxidative phosphorylation, dysregulation of calcium homeostasis, and elevated levels of sporadic mutations in the mtDNA in AD [41]. Low-dose treatment with MB can reduce ROS production, which could be beneficial to AD patients [43].

Several studies suggest that there is an association between mitochondrial dysfunction and abnormal processing of Aβ and tau [44,45,46,47]. Amyloid precursor protein (APP) can be trapped in the mitochondrial membrane and impair mitochondrial function [48]. Overexpression of tau can also result in mitochondrial dysfunction by decreasing ATP production and increasing oxidative stress [49]. Conversely, the damaged mitochondrial function can induce aberrant Aβ production and promote abnormal phosphorylation of tau [50]. MB was reported to prevent Aβ and tau aggregation or dissolve existing aggregates via autophagic clearance, and therefore alleviate downstream pathological consequences [51,52,53,54]. MB could directly or indirectly target β-secretase cleavage of amyloid precursor protein (APP) and regulate the generation of Aβ [55]. MB’s role in Aβ and tau aggregation clearance may help improve mitochondrial functions in AD neurons and thus contribute to AD treatment. Besides, cytochrome oxidase activity has been shown to decline in AD [56], while MB can increase the enzymatic activity of cytochrome oxidase, which results in an increased oxidative metabolic capacity of neurons [57].

The efficacy of MB in AD clinical treatment is still under investigation. In AD transgenic mouse models, MB can inhibit Aβ production and rescue the cognitive defects [58,59]. In 2008, a research group presented preliminary data showing that low-dose MB prevented cognitive impairment in AD patients. Rember, which is the commercial name of MB, was administered to patients with mild to moderate symptoms. The result showed that there was an 81% reduction in the rate of cognitive decline in 50 weeks [60]. In the phase 2 clinical trial, Rember was shown to improve both cognitive and cerebral blood flow in patients with mild to moderate AD [61]. LMTM, a stable variant form of MB, has been tested in a phase 3 clinical trial [62]. However, the result was inconclusive due to the lack of using the proper placebo control group [62]. Another non-randomized cohort analysis has been performed more recently, which showed that the brain atrophy rate in patients with mild AD declined after a 9-month treatment [63]. Still, treatment dosage and proper control group should be optimized, and a further suitably randomized trial is needed.

PD is another neurodegenerative disease associated with aging. It is one of the most common movement diseases featured by dopaminergic neuronal damage [64]. The pathological hallmark of PD is Lewy Bodies and Lewy Neurites, intracellular aggregates of the protein α-synuclein (α-syn) [65]. A-syn can lead to progressive mitochondrial dysfunction when translocated to the mitochondria [66]. Mitochondrial dysfunction is considered the primary cause of dopaminergic apoptosis via inducing oxidative stress in PD [67]. Based on MB’s role in improving mitochondrial function, MB could be a promising treatment in PD. Significant beneficial effects in reducing nigrostriatal dopaminergic loss and motor impairment can be observed in the rat rotenone models [68,69]. MB was able to preserve dopamine neurons to some degree and alleviate motor defects in a PD 6-OHDA mouse model [70]. Low-dose MB treatment is also effective in a chronic toxin-induced mouse model [32]. However, more evidence is still needed to confirm whether MB can be used as a PD treatment.

In addition to its beneficial role in age-related brain disorders, MB is a promising memory enhancer. Since metabolic derangement is observed in old brains, and mitochondrial impairment accumulates over time, improving mitochondria may help neurons maintain their health and improve their functions [71,72,73]. Several early studies have shown that MB could enhance memory retention by increasing cytochrome oxidase activity and facilitating ATP generation in rats and zebrafish at low dosages [34,74,75]. One of these studies showed that brain cytochrome oxidase activity after MB treatment was about 70% higher than in the control group, and the overall mnemonic capacity during discrimination learning was improved [74]. These effects were accompanied by long-lasting mitochondrial respiratory function [33]. Another study showed that MB could prevent memory impairment in rats with chronic cerebral hypoperfusion [76]. With functional imaging in the human brain, it was shown that MB could modulate task-related and resting-state neural networks [77]. Therefore, MB has the potential to protect cognition against accelerated aging. 

### 3.2. MB in Skin Aging

**Figure 2:**
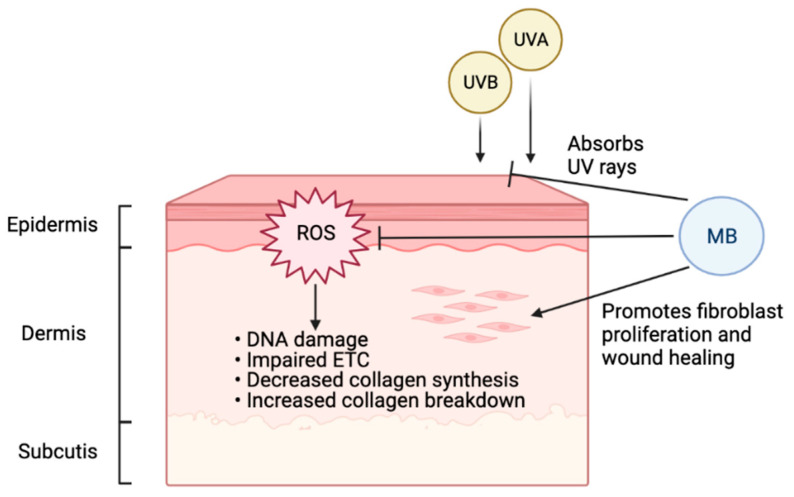
Beneficial effects of MB on human skin. Environmental factors, especially UV radiation, cause the accumulation of ROS on skin, leading to DNA damage, impaired ETC, decreased collagen synthesis, and increased collagen breakdown. As a potent antioxidant, MB can effectively combat ROS and presents the accelerated aging induced by ROS. In addition, MB can stimulate fibroblast proliferation, thereby promoting wound healing. Furthermore, MB is a broad-spectrum UV chemical blocker (Figure created with BioRender.com, 10 October 2021). Together, MB renders three levels of protection to human skin, from blocking UV irradiation, mitigating the cellular oxidative damages, to stimulating skin proliferation and wound healing.

As one of the body’s largest and most complex organs and the primary defense against the external environment, the skin serves a critical health function [78]. There are three primary layers of skin (Figure 2): the epidermis, the dermis, and the subcutis [79]. Keratinocytes and melanocytes localize to the innermost layer of the epidermis, called the basal cell layer [79]. The dermis contains fibroblasts, which synthesize extracellular proteins such as collagen and elastin [79]. The subcutis is also known as the hypodermis, the layer of subcutaneous fat that provides insulation [79]. 

Aging is an inevitable process that afflicts the skin. It is characterized by loss of elasticity, thinness, flattening of the dermal-epidermal junction, atrophy of the extracellular matrix (ECM), and ROS [80]. There are two different types of skin aging, intrinsic and extrinsic [81]. Intrinsic aging manifests in the natural physiologic factors: as the proliferation of the basal cell layer decreases, skin cells reach senescence, and collagen expression is downregulated [81]. Extrinsic skin aging expedites aging through environmental factors, causing thickening of the epidermis, decreased proliferation of basal cells, collagen downregulation, and increased oxidative stress [81]. A large fraction of extrinsic aging comes from photodamage accumulated by ultraviolet (UV) rays [82]. Oxidative stress is involved in both intrinsic and extrinsic skin aging [83]. Especially when skin is exposed to certain environmental risk factors such as UV radiation, increasing oxidative damage will decrease collagen synthesis and increase collagen breakdown, leading to accelerated aging [84]. Antioxidants such as MB can therefore protect skin and slow down the aging process (Figure 2).

A previous study has shown that MB treatment in normal fibroblasts could increase lifespan and cell proliferation while reducing aging markers [85]. In that study, MB increased cytochrome oxidase by 30%, enhanced oxygen consumption by 37–70%, and reversed premature senescence caused by H_2_O_2_ or cadmium, and the ratio MB/cytochrome c could be important for MB’s protective role. Interestingly, when MB was compared with common antioxidants used in skincare, including vitamin C and retinol (vitamin A), MB-treated skin cells outperformed both of them significantly in terms of promoting cell proliferation and reducing age-related markers [86,87]. In addition to its potent antioxidant function, MB treatment in skin fibroblasts could stimulate the expression of ECM proteins, including upregulation of elastin and collagen 2A1 [86]. Furthermore, MB treatment improved skin thickness and hydration in a 3D skin tissue model [86]. Our recent study showed that MB provides broad-spectrum absorption of UV rays and mitigates DNA double-strand breaks caused by UVB irradiation in human keratinocytes [87]. Together, this evidence supports MB as protective and beneficial to human skin, suggesting that the inclusion of MB in daily skincare may effectively delay photoaging.

MB can facilitate wound healing. During aging, the proliferation and migration of fibroblasts are often decreased, and collagen and elastin in the ECM are degraded [88]. Therefore, the repair capabilities of the skin decline due to structural and functional changes. Our study indicated that MB treatment could promote fibroblast migration and proliferation in the wound healing process [86]. In skin survival burn models in rats, MB treatment could reduce necrosis progression, which might be mediated by decreasing oxidative stress through blocking nitric oxide (NO) [89]. Moreover, MB can facilitate wound healing by reducing antimicrobial burden and decreasing hyper-granulation. MB also has a drying effect without harming healthy cells [90,91,92]. Overall, MB was shown to improve tissue viability with little to no irritation in laboratory models. 

### 3.3. MB in Progeria

MB has shown potential for the treatment of Hutchinson-Gilford Progeria Syndrome (HGPS), which is a genetic, premature aging disorder caused by a C to T *de novo* point mutation on exon 11 of the *LMNA* gene [93]. The mutation causes the production of progerin in the place of Lamin A, which holds a cryptic splice site that results in a 50 amino acids deletion of the protein [93]. The deletion removes a critical protease cleavage site, making progerin an aberrant form of pre-Lamin A with a permanent farnesyl modification. Progerin is then localized to the nuclear envelope, where it becomes fixed in the nuclear membrane [93]. Progerin disrupts the nuclear skeleton, leading to nuclear abnormalities, blebbing of the nuclear envelope, transcriptional changes, and severe cellular stress, which ultimately manifest as a rapid aged phenotype in patients [94,95,96]. Mitochondrial dysfunction is observed in both HGPS fibroblasts and animal models, with elevated levels of mitochondrial-specific superoxide (MitoSOX), ROS, and suppression of the mitochondrial biosynthesis gene PGC-1α [97,98].

As a mitochondrial antioxidant and highly permeable molecule, MB has demonstrated the ability to rescue HGPS phenotypes [99]. Our group found that MB treatment not only reduced mitochondrial defects but also rescued the transcriptional changes in HGPS. Besides, MB demonstrates the ability to solubilize progerin in the nuclear envelope, correcting the blebbed nuclei of HGPS cells and drastically reducing cellular stress. Whether these effects are related to MB’s antioxidant characteristic is still unclear.

## 4. Discussion

Aging is a complex process with multiple contributing factors. This review discussed the anti-aging effects of MB with a focus on the relationship between mitochondrial dysfunction and aging. Brain aging is a mystery and neurodegenerative disorders are a major part. Currently, no cure is fully developed for most neurodegenerative diseases, including AD and PD. Several observations suggest that mitochondrial dysfunction is a key pathogenic step in neurodegenerative conditions [100]. In addition to having antioxidative properties, MB also crosses the BBB easily, making it a promising candidate for treatment. In AD mouse models, low-dosage MB shows its ability to relieve oxidative stress and rescue cognitive defects [58,59]. However, the results in phase 2 and phase 3 clinical trials are controversial [62,63,101]. This could be due to the translational limitations of mouse models. The design of clinical trials needs to be further optimized. Moreover, several studies suggest that MB can alleviate behavioral defects in PD mouse models [70]. Studies show that, even with physiological brain aging, MB treatment could still enhance memory by increasing cytochrome oxidase activity and decreasing oxidative stress [52,53,54,55,56]. However, more extensive studies are required to validate the clinical applications of MB in brain aging. MB has the ability to delay skin aging as well. MB can increase cell longevity, protect skin from UV exposure, and accelerate and help with the wound healing process [86,87]. Additionally, MB’s antimicrobial properties are beneficial for skin [79,80,81]. Furthermore, a recent study proved that MB treatment resulted in significant improvements in HGPS skin fibroblast phenotypes (an accelerated form of the aging disease) [99].

Since MB is an FDA-approved medicine with a long history, the safety of MB usage has been thoroughly evaluated. The exploration of MB utilization in aging-related conditions can help us understand the aging process. Derivatives of MB can also be developed to improve its effects.
